# The Treatment of Snake-Bites

**Published:** 1890-05

**Authors:** 


THE TREATMENT OF SNAKE-BITES. DR. S. WEIR MITCHELL'S UNDIGNIFIED DIG AT HOMŒOPATHY. By Wm. B. Clarke, M. D., Indianapolis, Indiana, Secretary of Indiana Institute of Homœopathy.

This is a pamphlet of seven pages containing an answer to the slurring allusion to Homœopathy contained in Dr. Mitchell's article upon "The Poison of Serpents," in the *Century Magazine* last August. In the course of his answer, Dr. Clarke shows that alcohol is not a perfectly reliable remedy for snake-bite, but that in California there grows a weed, a species of the plant euphorbia, which is a safe remedy.

He also shows that venomous snakes carry in their gall bladder an antidote to their own poison. The Lachesis poison may be antidoted with tincture of Aristolochia Columbiana. To the poison of the rattlesnake there is said to be an antidote in a common plant called Hyssop.

A strong alkali will, when applied without delay to a snake-bite, destroy the venom. Hence the value of Ammonium-carbonate, the dry powder of which should be stuffed into the wound and small dissolved doses taken internally.

The local application of crystals of Permanganate of Potassa, preferably by injection, will kill the venom in a snake-bite. This is the proposition of Dr. Lacerda, of Rio Janeiro, and the Emperor of Brazil gave him a present of $25,000 in gold for his discovery. W. M. J.

